# Sex differences in recovery of motor function in a rhesus monkey model of cortical injury

**DOI:** 10.1186/s13293-021-00398-9

**Published:** 2021-10-09

**Authors:** Karen R. Bottenfield, Bethany G. E. Bowley, Monica A. Pessina, Maria Medalla, Douglas L. Rosene, Tara L. Moore

**Affiliations:** 1 Dept. of Anatomy & Neurobiology, Boston University School of Medicine, 700 Albany Street, W701, Boston, MA 02118 USA; 2 Center for Systems Neuroscience, Boston University, Boston, MA 02215 USA

**Keywords:** Motor cortex, Injury, Stroke, Recovery, Sex, Estrogen, Rhesus, Monkey, Ischemia, Hand

## Abstract

**Background:**

Stroke disproportionately affects men and women, with women over 65 years experiencing increased severity of impairment and higher mortality rates than men. Human studies have explored risk factors that contribute to these differences, but additional research is needed to investigate how sex differences affect functional recovery and hence the severity of impairment. In the present study, we used our rhesus monkey model of cortical injury and fine motor impairment to compare sex differences in the rate and degree of motor recovery following this injury.

**Methods:**

Aged male and female rhesus monkeys were trained on a task of fine motor function of the hand before undergoing surgery to produce a cortical lesion limited to the hand area representation of the primary motor cortex. Post-operative testing began two weeks after the surgery and continued for 12 weeks. All trials were video recorded and latency to retrieve a reward was quantitatively measured to assess the trajectory of post-operative response latency and grasp pattern compared to pre-operative levels.

**Results:**

Postmortem analysis showed no differences in lesion volume between male and female monkeys. However, female monkeys returned to their pre-operative latency and grasp patterns significantly faster than males.

**Conclusions:**

These findings demonstrate the need for additional studies to further investigate the role of estrogens and other sex hormones that may differentially affect recovery outcomes in the primate brain.

## Highlights

- Aged female and male rhesus monkeys were trained on a fine motor task before undergoing surgery to produce a lesion to the hand area representation of the primary motor cortex
- Aged female monkeys returned to pre-operative latency and grasp patterns faster than aged males after cortical injury

## Background

According to Framingham Heart Study data, 1 in 5 women over the age of 55 will experience a stroke in their lifetime [1, 2]. Further, it has been established that females experience more severe strokes and have a greater functional impairment and higher mortality rate than males [2–7]. However, prior to advanced age (~ 65 years), the incidence of stroke is higher in males than in females [1, 8]. This has been attributed to the neuroprotective effects of sex hormones, especially estradiol (E2), which is present in higher levels in young compared to aged females [9]. For example, Alkayed et al. [10] showed that young female rats with normal physiological levels of 17 beta-estradiol (17ß-E2) sustained less neuronal damage following stroke than young males. Further, middle cerebral artery occlusion (MCAOs) in spontaneous hypertensive female rats during proestrus periods (high circulating estradiol) resulted in less cortical damage than MCAO during metestrus (low circulating estradiol) [11]. Also, infarct size in female animals pre-treated with 17ß-E2 prior to MCAO was more than 50% smaller than in animals without pre-treatment [12–14]. These studies and many others have established that estradiol plays a role in reducing infarct size and improves functional outcome following ischemia.

Estradiol has anti-inflammatory properties and contributes to plasticity following injury [15–23]. Estradiol acts on ER-α and ER-β receptors expressed on neurons, astrocytes, and microglia to modulate the inflammatory response and promote repair cascades in rodents [13, 16, 17, 19, 24, 25]. Progesterone has also been attributed to neuroprotection in animal models of brain damage [26–28]. For example, administration of progesterone to ovariectomized female and male rodents resulted in reduced infarct volume and improved functional recovery after MCAO [29–34]. Progesterone receptors (PRs) are broadly expressed by neurons and glia and modulate the release of pro-inflammatory cytokines and reduce oxidative stress [28, 35–38]. A large body of evidence from studies in rodents and in vitro cultures demonstrates sex differences in the immune cells and oxidative stress pathways mediating these inflammatory and repair responses after injury [13, 22, 24, 39, 40]. However, how these sex differences affect the precise nature and degree of recovery after cortical injury is not well understood in the aged primate brain.

Despite considerable evidence of the effect of estradiol and progesterone on recovery and inflammation following cortical injury [15–19, 26, 36, 41–46], there have been limited studies looking at cortical injury and recovery in the female brain in a higher order, gyrencephalic animal species. Further, while epidemiological studies in humans have shed light on sex differences in the clinical risk and severity of stroke, variability in the human datasets precludes precise quantitative studies and establishment of predictive models of how sex affects severity of functional impairments and degree of recovery after injury. Our group has successfully established a rhesus monkey model of cortical injury [47] in which we determined the degree of impairment and the rate and pattern of recovery of function following cortical injury in young and middle-aged male rhesus monkeys. In this model of cortical injury, a targeted lesion is made in the hand representation of the primary motor cortex to quantify the extent of impairment and subsequent progression of recovery of fine motor hand movement [47–51]. In the present study, using this model, we now compare the rate and pattern of recovery of function following cortical injury in aged male and female monkeys. Data from this study provide insight into the rate and extent of recovery in response to cortical injury in the female monkey brain compared to the male rhesus monkeys.

## Methods

### Animals

Four male and five female aged rhesus monkeys (Macaca *mulatta*) (16–26 years, equivalent to approximately 48–78 year-old humans) [52] were used in this study. Monkeys were obtained from national primate research facilities or private vendors and had known birth dates and complete health records. Monkeys received medical examinations and magnetic resonance imaging to ensure there was no occult health problems or neurological damage. Monkeys were housed in the Animal Science Center of Boston University School of Medicine which is AAALAC accredited. All procedures were approved by the Boston University Institutional Animal Care and Use Committee.

### Pre-operative training on fine motor function test

**Fig. 1:**
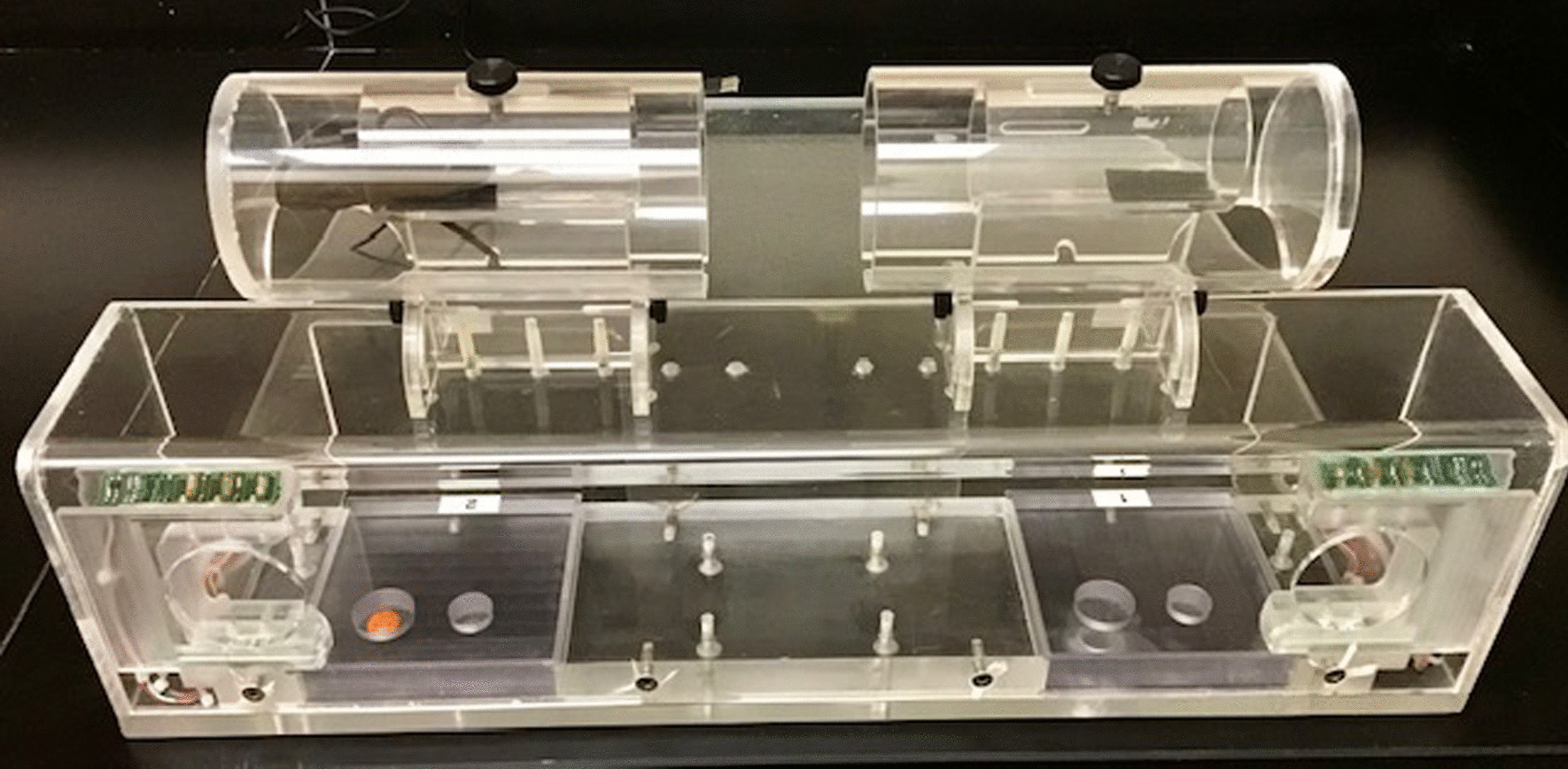
Monkey view of the testing apparatus for the HDT. Two openings, one on each side of the testing apparatus, allowed the monkey to reach through and retrieve the food reward from a baited well in a Plexiglas tray. A timer is connected to photocells located in the openings for recording of time to retrieve the food reward. Retrieval is also recorded by fixed placement cameras mounted above the apparatus for assessment of grasp patterns

As described in detail previously [49, 53], monkeys were trained on a task of fine motor function of the hand, the Hand Dexterity Task (HDT), using a testing apparatus that controls, quantifies and video records responses from each hand (Fig. 1). Using this apparatus, monkeys were trained on the HDT for a total of 12 days (Monday, Wednesday, and Friday each week for 4 weeks). The HDT is a modified version of a Klüver board [54] and requires precise control of the digits, particularly apposition of the thumb and index finger, to efficiently retrieve a small, visible food reward (M&M’s, Mars, Inc.) from two different size round wells in a Plexiglas tray. Food rewards were round and approximately 1 cm in diameter. Both wells were 1 cm deep. The large well was 25 mm wide and the small well was 18 mm wide. Time to retrieve the food reward was recorded by a timer connected to photocells located in the openings on each side of the apparatus that allowed hand access to the baited wells. The timer starts when the monkey puts a hand through the opening, triggering the photocells to start the timer. The timer stops when the monkey removes its hand. An experimenter records whether or not the reward is successfully retrieved and the response time to retrieve is recorded. The HDT has been used to assess fine motor function of the hand and digits in adult monkeys with and without injury to the hand representation in the motor cortex, as well as to compare the performance of middle-aged rhesus monkeys to young adult monkeys [47, 53]. Each test day consists of 16 trials for each of the two well sizes and for each hand, resulting in a total of 32 trials. The order of trials for each hand and well follows a pseudorandom balanced sequence to eliminate any order effects. Monkeys were given 30 s to complete a trial. If they did not complete a trial in 30 s, the trial was terminated, and the monkey was given one additional opportunity to complete that trial. After a second failed attempt, a non-response was recorded, the monkey’s difficulties were noted in the study record, and the next trial was initiated.

### Hand preference

At the completion of pre-operative training on the HDT, free choice trials with both sides of the apparatus baited and accessible were administered to determine which hand was “preferred”. This assessment was also compared with the pre-operative response latencies for each hand. Based on this assessment, the cortical injury was targeted to the hand representation of the hemisphere controlling the preferred hand. This ensured that monkeys were motivated to use the impaired hand during post-operative testing.

### Electrophysiological mapping of the hand representation in motor cortex

All surgical procedures were carried out under aseptic conditions. Description of the surgical procedure was reported in detail in Moore et al. 2019. In brief, the head was stabilized in a stereotactic apparatus, and a midline incision made followed by reflection of the temporalis muscle. A bone flap approximately 40 mm in anterior to posterior extent and 35 mm in medial to lateral extent was made centered over the precentral gyrus. The bone was removed in one piece and replaced at the end of surgery. The dura was incised to expose the precentral sulcus and primary motor cortex.

**Fig. 2:**
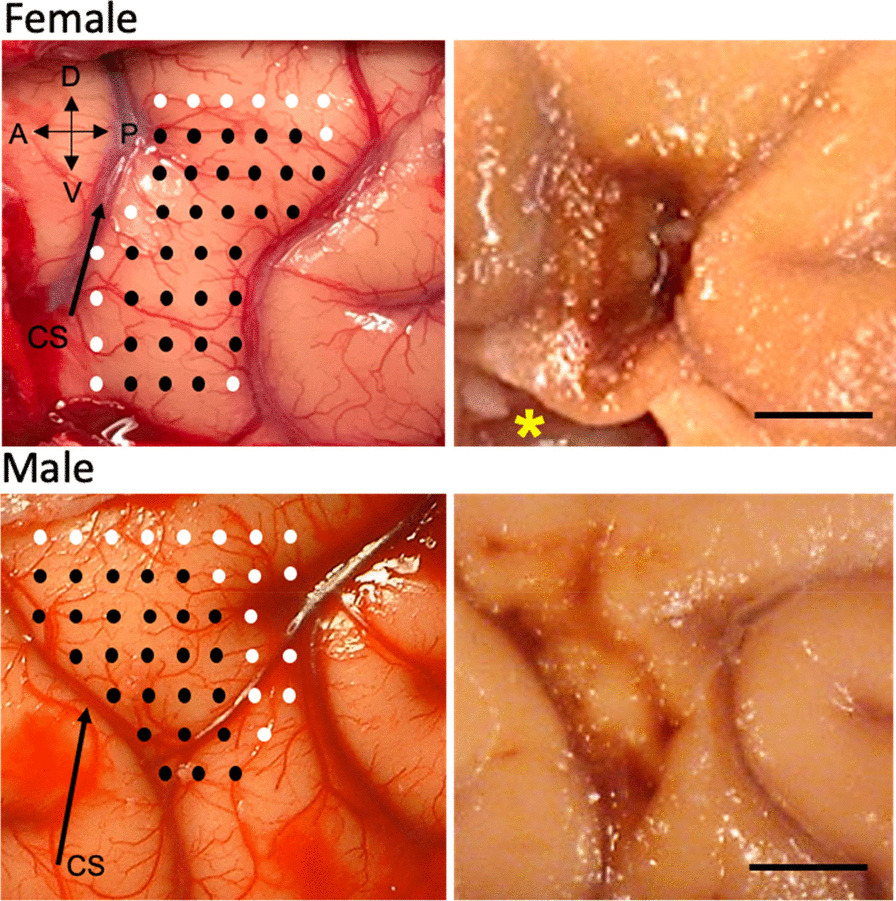
Representative photographs of cortical maps and lesions. Photos showing the stimulation sites on the hand representation maps and the final lesion after perfusion for one female monkey (top row) and one male monkey (bottom row). On the hand representation maps, the black circles represent stimulation sites that generated a positive response in the hand or digits and the white circles represent stimulation sites that did not generate a positive response. *area where a fresh tissue sample was extracted at perfusion. *CS* Central Sulcus. Scale bar = 5 mm

To create reproducible cortical injury and motor deficits limited to the hand, a calibrated photograph of the pre-central gyrus was taken and printed. The precentral gyrus was then systematically explored using electrical stimulation delivered through a small monopolar silver ball electrode placed gently on the surface of the pia to evoke movements. A surface electrode was used rather than a sharp electrode that penetrates the cortex in order to avoid extraneous damage to the motor cortex outside the hand representation. The stimulating electrode was moved across the precentral gyrus systematically in rows spaced approximately 2 mm apart ventral to dorsal with each stimulation site in the row separated by 2 mm in anterior to posterior direction as shown in Fig. 2. Monopolar stimulus pulses of 250 µsec duration at amplitudes from 2.0 to 3.0 mA were delivered at each site once every 2 s first singly and then in a short train of 4 pulses at a rate of 100 Hz. Non-responsive sites were further tested with a 200 Hz train consisting of 4 or 8 pulses of 2 ms duration. During each stimulation, a trained observer noted muscle movement (e.g. distinct movement or twitches of muscle) in specific areas of the digits, hand, forearm or arm, both visually and by palpation. The intensity of the motor response in the hand and digits was graded on a scale of 1 to 3 (barely visible to maximal). Specific stimulation sites with the lowest threshold and highest motor response were marked on the calibrated photograph creating a cortical surface map of the hand area that was used to guide placement of the lesion (Fig. 2).

### Placement of selective cortical injury

Using the map described above, cortical injury was induced by making a small incision in the pia at the dorsal limit of the mapped representation. A small glass suction pipette was then inserted under the pia and used to bluntly transect the small penetrating arterioles as they leave the pia and enter the underlying cortex. Suction and irrigation with sterile saline are sufficient to stanch any bleeding and maintain a clear field. Since the hand representation is known to extend down the rostral bank of the central sulcus, the central sulcus was opened along the length of the gyral hand representation and exposed down to the fundus by microdissection with a small glass pipette and a blunt periosteal elevator. As on the surface, the pia was then dissected with the glass pipette down to the fundus on the rostral bank of the sulcus taking care to leave the somatosensory cortex on the caudal bank intact. The hand area in the sulcus was not electrophysiologically mapped with the electrode to avoid inadvertent damage to the somatosensory as this mapping would require prolonged retraction. However, we have verified electrophysiologically in terminal experiments the presence of the hand representation on the rostral bank beneath the gyral representation. The pial dissection of penetrating vessels removes the blood supply to the cortex of the hand representation, inducing damage that extends down to the underlying white matter. Representative photos of the cortical map and lesion for one male and one female monkey are shown in Fig. 2.

### Post-operative testing

Post-operative testing on the HDT began two weeks after surgery and continued for 12 weeks. As in preoperative testing, this was conducted on Monday, Wednesday, and Friday of each week. However, post-operatively 70% of the trials were given to the side of the impaired hand, while 30% were given to the intact hand. The 30% of trials given to the unimpaired hand provided sufficient rewards to maintain motivation and sufficient data to demonstrate that the effects were not due to generalized changes in motivation or motor function. The forced use of the impaired hand on 70% of the trials is similar in nature to constraint-induced therapy used in human rehabilitation which forces use of the impaired limbs [55–57]. Each monkey was given 30 s to complete a trial as in pre-operative training and this continued for 12 weeks. This time point was chosen as performance on the HDT by monkeys with cortical injury reaches a stable asymptotic level at this time.

### Grasp pattern assessment

While some spontaneous recovery does occur after injury to cortical motor areas controlling the hand and digits, full recovery of digit function is rare. Further, the spontaneous recovery that does occur is largely compensatory in nature and involves mass action of the entire hand rather than a return to pre-injury fine motor function [58]. The development of compensatory grasp falls short of full functional recovery as it still limits normal activities of daily living so the distinction between complete and compensatory recovery is important for assessing new treatments for recovery from stroke or other cortical injury [59, 60]. To address this, we developed a Non-Human Primate Grasp Assessment Scale (GRAS) to detect and quantify impairments in fine motor function of the hand and evaluate recovery of function of individual digits and precise finger-thumb pinch used to retrieve food morsels. The GRAS allows us to objectively distinguish between compensatory grasp function and a return to pre-injury fine motor grasp patterns [61].

This GRAS was adapted from the Eshkol-Wachman Movement Notation [62, 63] and the Fugl-Meyer Motor Assessment scale [64]. It rates the position of the digits during grasp and the pattern of grasp and release to provide a semi-quantitative measure of maturity of the grasp pattern. The scale includes 8 divided hierarchical stages, for a total of 14 units with the maximum score of 8 reflecting normal grasp patterns (functional pinch between thumb and one individual digit) [61]. To apply the GRAS scale to our monkeys, performance on the HDT during pre-operative training and post-operative testing was recorded with fixed placement cameras (Logitech, Newark, CA). A licensed Occupational Therapist (MAP) who has clinical experience in the treatment of patients with upper extremity impairment following stroke, and a trained research technician (BGEB) analyzed the video recordings using our NHP Grasp Assessment Scale.

### Perfusion and lesion assessment

At the end of the 12 week post-operative period, monkeys were deeply anesthetized with IV sodium pentobarbital (25 mg/kg to effect) and euthanized by exsanguination during transcardial perfusion of the brain, first for no more than 5 min with 4 °C Krebs buffer at pH 7.4 and then with 8 L of 37 °C 4% paraformaldehyde, (pH 7.4) over 10 min to completely fix the brain. The skull was opened and the brain was photographed in situ with the photograph aligned to the perspective of the cortical map used to create the lesion. The brain was blocked in situ in the coronal plane to ensure reproducible planes of section during later processing. The brain was removed from the skull, weighed and post-fixed overnight in 4% paraformaldehyde for no more than 18 h. To eliminate freezing artifact, the brain was then transferred to cryoprotectant solutions of glycerol and buffer and flash frozen at -75° C and stored at – 80 °C [65]. Frozen blocks were later removed from storage and cut on a sliding microtome into interrupted series of coronal sections (eight series of 30 μm thick sections and one 60 μm thick series, with 300 μm spacing between sections). The 60 μm series was immediately mounted on microscope slides and stained with thionin for lesion reconstruction. Other series were collected in buffer with 15% glycerol, equilibrated overnight at 4 °C and stored at – 80 °C for later histochemical processing [66]. Lesion volume was determined for all of the monkeys as described in Go et al. [67] and Moore et al. [47].

## Results

### Pre-operative performance

To establish baseline performance, the mean latency to retrieve across the last 5 days of pre-operative training was calculated for each subject. There was no significant difference between the male and female monkeys in performance on the small well [*F* (1, 8) = 0.396, *p* = 0.549] or the large well [F (1, 8) = 0.145, *p* = 0.7189] (Table 1).

**Table 1:** Subject data and individual data on sex, hand preference, age, lesion volume, and pre-operative latency (measured in seconds)

Monkeys	Sex	Hand preference	Age at surgery	Lesion volume (mm^3^)	Mean pre-operative latency—large well	Mean pre-operative latency—small well
AM323	F	L	23.67	34.03	1.10	1.15
AM331	F	R	26.08	52.18	2.20	1.77
AM335	F	R	20.33	25.81	1.53	1.25
AM337	F	L	24.33	25.83	0.89	1.08
AM339	F	R	21.42	31.27	1.41	1.07
Mean			23.17	33.82	1.43	1.26
SE			1.03	4.86	0.22	0.13
SM006	M	L	17.17	27.83	1.82	1.75
SM007	M	L	17.00	68.65	2.11	1.97
SM010	M	L	21.08	59.48	0.99	1.10
SM027	M	L	12.00	88.07	1.29	0.91
Mean			16.81	61.01	1.55	1.43
SE			1.66	11.24	0.25	0.25

### Post-operative latencies in females compared to males

**Fig. 3:**
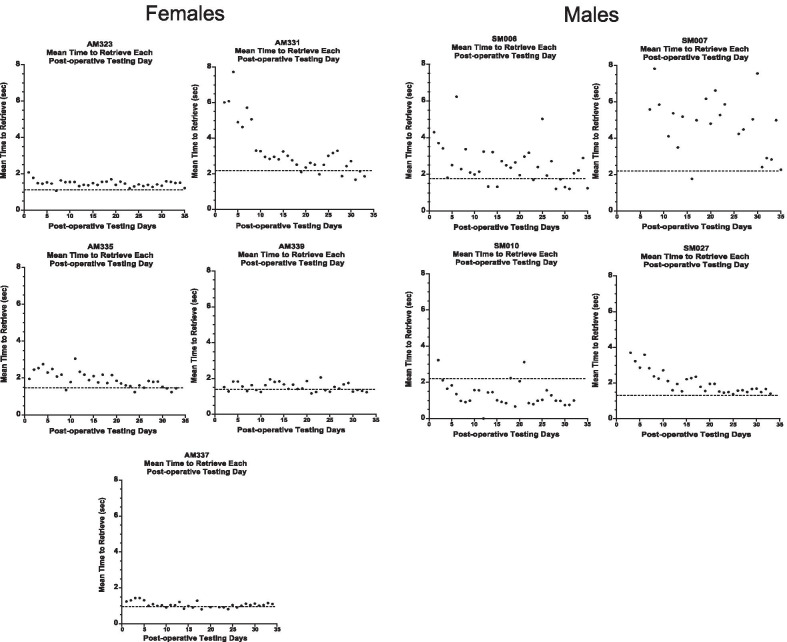
Mean time to retrieve the food reward each day during the 12 week post-operative period. Graphs of the daily mean time to retrieve a food reward from the large well with the impaired hand across the post-operative recovery period. The dashed line represents the mean time to retrieve the food reward over the last five days of the pre-operative training. Each data point (black dots) represents the mean time to retrieve for each post-operative day

Figure 3 shows the mean time to retrieve the food reward each day during the entire 12 week post-operative testing period for each monkey with the dashed line on each graph delineating the pre-operative baseline.

**Fig. 4:**
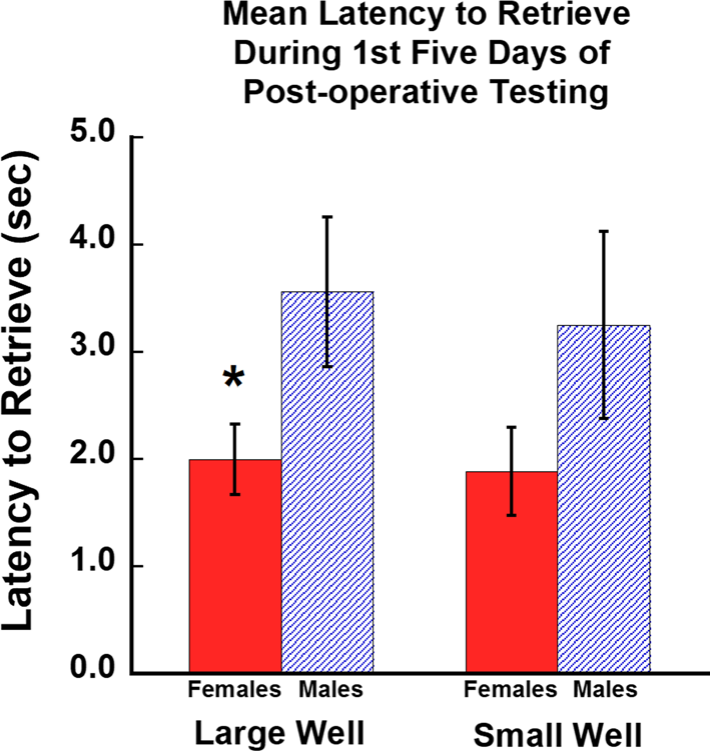
Mean latency to retrieve during the first five days of post-operative testing. Graph of mean latency to retrieve the food reward from each of the two wells (large and small) during the first five days of post-operative testing. Female monkeys retrieved the reward at a faster rate than males during this early post-operative period on the large well. Error bars = Standard Error, *p = 0.04

As shown in Fig. 4, the mean latency to retrieve the food reward during the 1^st^ five days of post-operative testing (3rd week post-injury) was analyzed with a one-way ANOVA for the effect of sex. At this very early stage in the post-operative recovery period, female monkeys retrieved the food reward at a faster rate than male monkeys on the large well [*F* (1, 8) = 5.75, *p* = 0.04], but not the small well [*F* (1, 8) = 2.70, *p* = 0.144]. However, at the end of the 12-week post-operative testing period, eight of the nine monkeys had not returned to their baseline pre-operative latencies to retrieve.

**Fig. 5:**
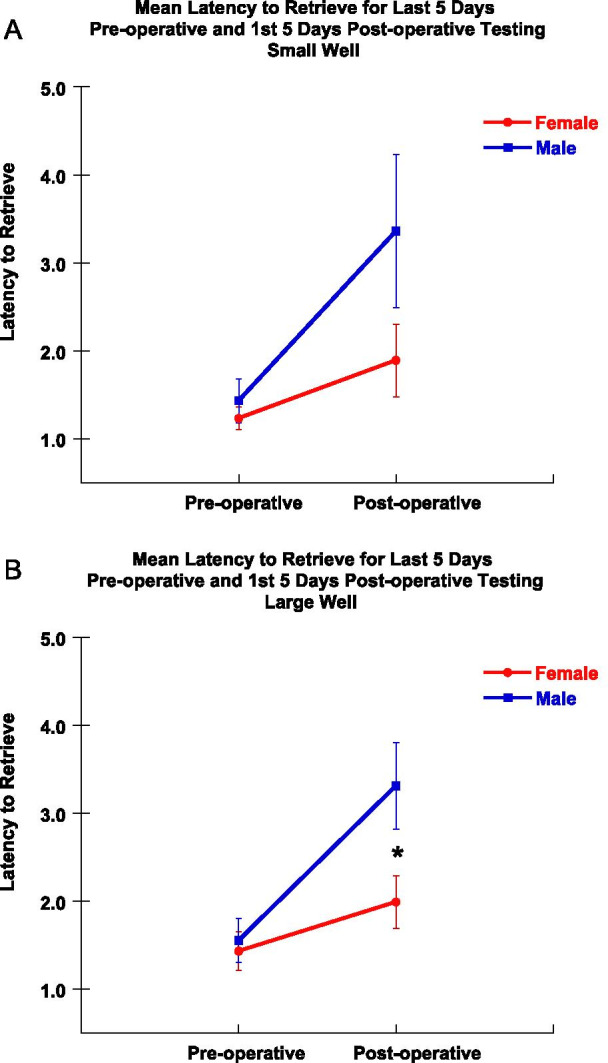
Female monkeys experienced less severe deficits in latency to retrieve after injury than males. **A** Graph of latency to retrieve for the last 5 days of pre-operative testing and the first 5 days of post-operative testing on the small well for male and female monkeys. Error bars = Standard Error. **B** Graph of latency to retrieve for the last 5 days of pre-operative testing and the first 5 days of post-operative testing on the large well for male and female monkeys. Error bars = Standard Error, *p = 0.01

In addition, as shown in Table 2, the difference in performance on the first 5 days of post-operative testing relative to the last 5 days of pre-operative testing, showed that female monkeys were significantly less impaired in latency to retrieve than the male monkeys after injury on the large well [*F* (1, 8) = 11.20, *p* = 0.01], but not on the small well [*F* (1, 8) = 2.97, *p* = 0.128] (Fig. 5) early in the recovery period. Taken together, these findings suggest that overall female monkeys experienced less severe deficits in latency to retrieve a food reward after injury than male monkeys.

**Table 2:** Mean latency data for each monkey during the post-operative period. Latency measured in seconds

	1st five days of post-operative testing		Difference between last 5 days pre-operative and first 5 days post-operative testing		All 12 weeks testing	
Monkeys	Mean latency—large well	Mean latency—small well	Mean latency—large well	Mean latency—small well	Mean latency—large well	Mean latency—small well
AM323	1.62	1.67	0.52	0.52	1.46	1.46
AM331	3.10	3.49	0.9	1.72	2.64	2.62
AM335	2.40	1.72	0.87	0.47	1.92	1.55
AM337	1.28	1.42	0.39	0.34	1.00	1.04
AM339	1.60	1.16	0.19	0.09	1.52	1.19
Mean	2.00	1.89	0.57	0.63	1.71	1.57
SE	0.33	0.41	0.14	0.28	0.27	0.28
SM006	3.53	3.19	1.71	1.44	2.42	2.58
SM007	5.42	5.60	3.31	3.63	6.00	4.76
SM010	2.03	1.37	1.04	0.27	1.45	1.33
SM027	3.25	2.85	1.96	1.94	2.05	1.62
Mean	3.56	3.25	2.01	1.82	2.98	2.57
SE	0.70	0.88	0.48	0.70	1.03	0.78

### Post-operative grasp assessment rating in females compared to males

**Fig. 6:**
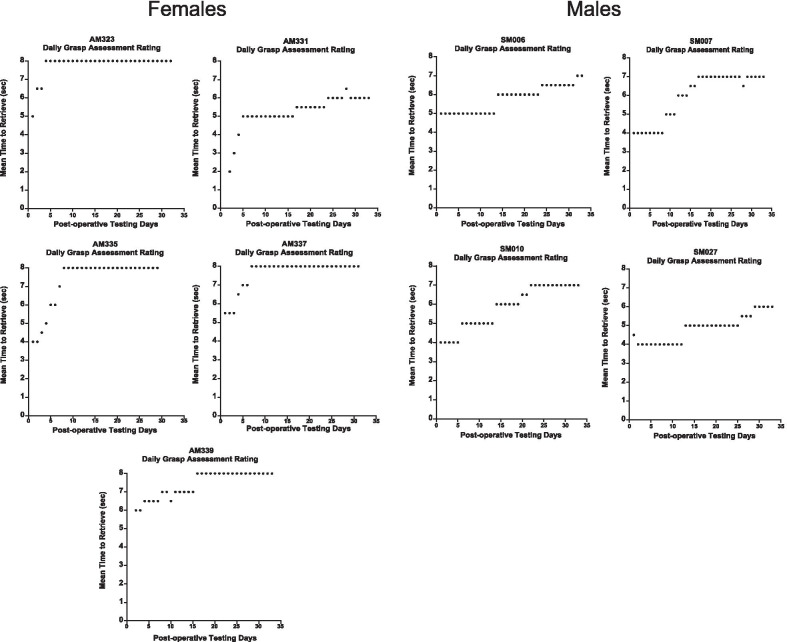
Mean grasp rating each day during the post-operative period. Graphs of the daily mean grasp rating of the impaired hand across the post-operative recovery period for each monkey. A score of 8 represents a normal grasp pattern that was documented during pre-operative training. Each data point (black dots) represents the mean grasp pattern score for each post-operative day

**Fig. 7:**
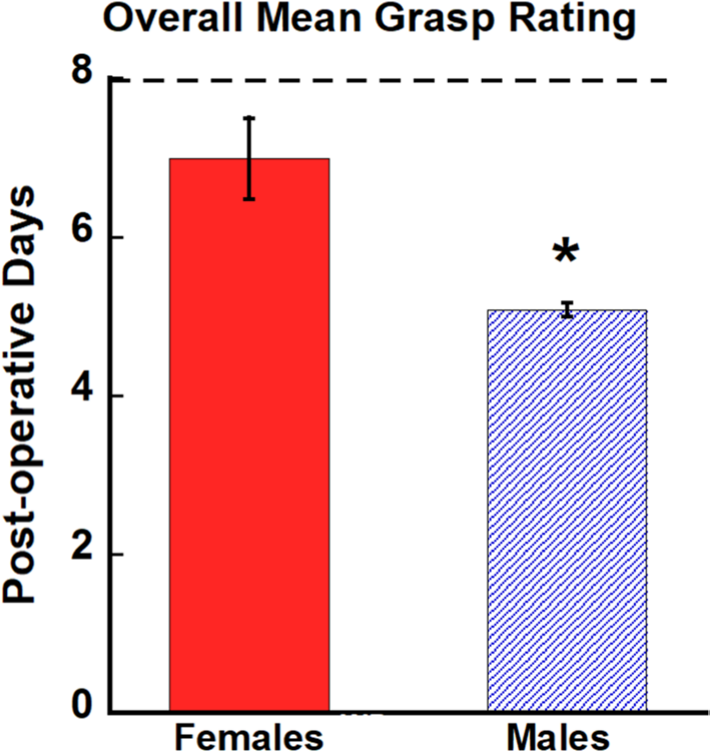
Graph of the mean grasp rating for each group. Female monkeys achieved an overall higher grasp rating than male monkeys. A score of 8 represents a return to pre-operative grasp patterns (dashed line). Error bars = Standard Error, *p = 0.004

While the latency to retrieve a food reward is an important measure of recovery, the quality of grasp demonstrated by the monkeys is critical for assessing human recovery of function as return to pre-operative grasp function will better allow for successful completion of activities of daily living than a compensatory grasp pattern. In order to determine the extent of recovery of pre-operative grasp in males versus females, we used our Non-Human Primate Grasp Assessment Scale (GRAS) to quantify fine motor function of the hand and to evaluate recovery of function of individual digits and precise finger-thumb pinch used by monkeys to retrieve food rewards. Figure 6 shows the mean grasp rating each day during the post-operative testing period for each monkey (a score of 8 represents a return to pre-operative grasp patterns). A one-way ANOVA compared the highest level of grasp rating achieved by the monkeys and revealed a significant difference between males and females, with female monkeys achieving an overall higher rating than the male monkeys [*F* (1, 8) = 17.91, *p* = 0.004] (Fig. 7). Furthermore, four of the five female monkeys returned to their pre-operative grasp pattern (a score of 8 on the GRAS) while none of the male monkeys returned to their pre-operative grasp, but instead demonstrated only compensatory grasp patterns.

### Fewer days to return to pre-operative grasp patterns in females compared to males

**Fig. 8:**
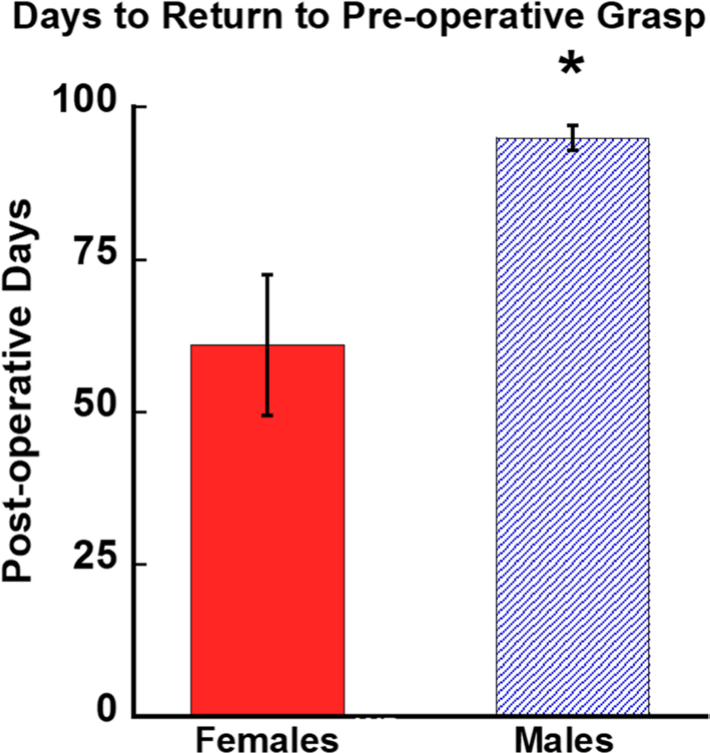
Graph of the mean number of days to return to pre-operative grasp patterns for each group. Female monkeys reached their highest grasp rating in fewer days than male monkeys. Error bars = Standard Error, *p = 0.04

Figure 8 shows the mean numbers of post-operative testing days for monkeys to return to pre-operative grasp patterns (a score of 8 on the GRAS) or to reach an asymptotic level of performance (three consecutive testing days at the highest achieved rating). A one-way ANOVA revealed that the female monkeys reached their highest rating in significantly fewer days than the male monkeys [*F* (1, 8) = 6.67, *p* = 0.04].

**Fig. 9:**
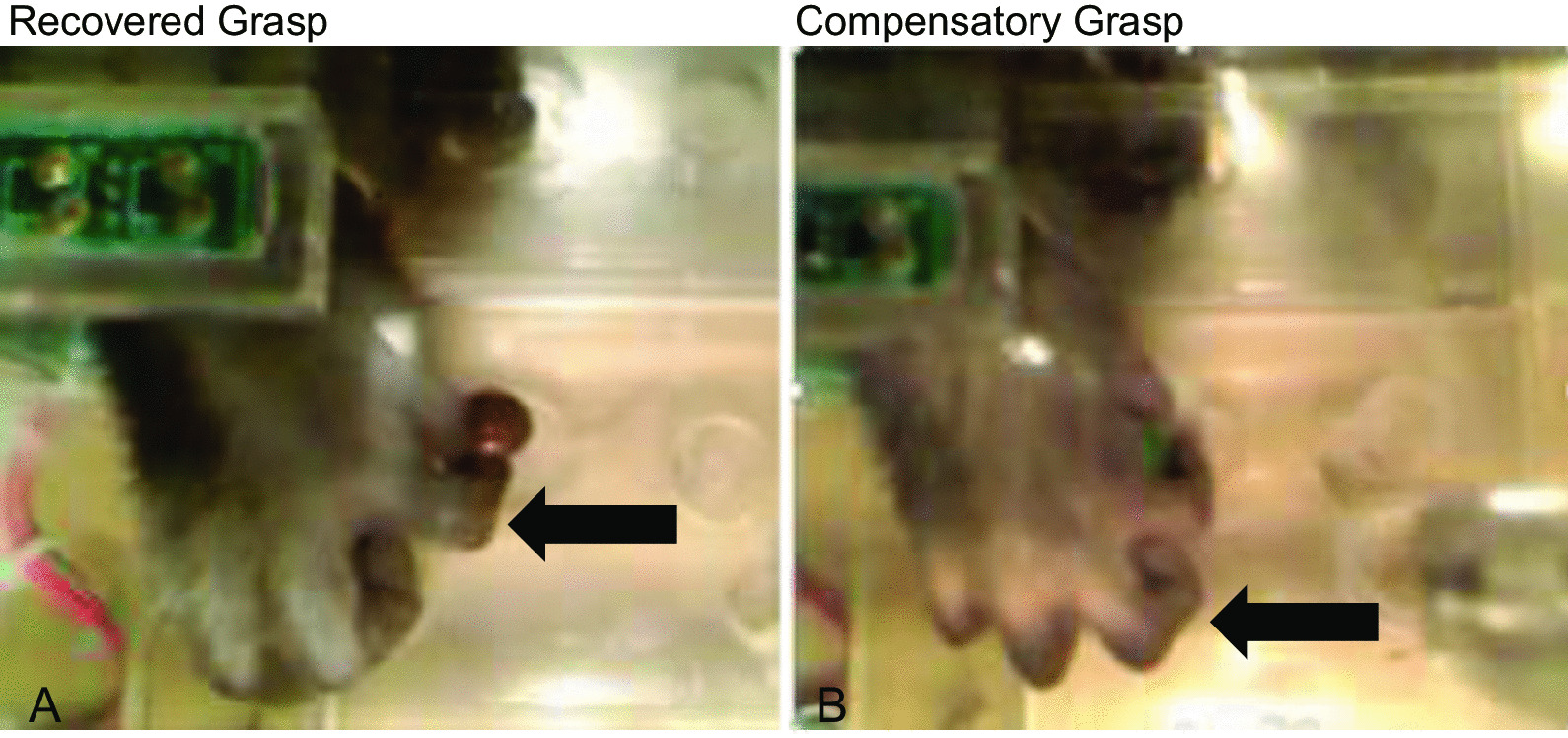
Representative images of post-operative grasp patterns. **A** Image shows recovered finger-thumb grasp of a monkey with isolated digit action and no evidence of “mass action” of the digits or compensatory scooping. Arrow in A shows precise finger-thumb grasp. **B** Image shows compensatory “scooping” involving all fingers of a monkey. The fingers work together to retrieve the food reward, a grasp that is referred to as “mass action” of digits. Arrow in B shows fingers scooping candy into the palm of the hand

Representative images in Fig. 9 show digit use of the impaired hand from one female monkey (showing the use of a finger-thumb pinch pre-operative grasp) and one male monkey (showing a compensatory grasp). Panel A shows a precise finger-thumb grasp that is representative of a greater degree of recovery of function. This grasp shows isolated digit action and no evidence of “mass action” of the digits or compensatory scooping. Panel B shows compensatory “scooping” involving mass action of all fingers working together to retrieve the food reward and is considered a compensatory grasp pattern. The arrow in panel B shows fingers scooping candy into palm of the hand.

It is of interest to note that during post-operative testing, retrieval from the large well is more difficult than retrieval from the small well due to the fact that, in general, the compensatory scooping grasp is more effective in scooping the reward out of the smaller diameter well. The monkeys are able to steady the treat on the side of the well and then scoop it into their palm. In the larger diameter well, this compensatory movement was more difficult in the larger space as the reward would ‘‘slip’’ away from the monkey while trying to scoop it out of the well without effective finger–thumb apposition. Therefore, the significant difference in performance between the male and female monkeys on the large well, further supports that the female monkeys were less impaired following injury to the hand representation of the primary motor cortex. Specifically, the male monkeys developed a compensatory grasp while the female monkeys returned to pre-operative grasp patterns.

### No significant sex differences in lesion volume

A one-way ANOVA was used to compare the volume of the lesion between the male and female monkeys. Results revealed no significant differences between groups [*F* (1, 8) = 4.86, *p* = 0.07] (Table 1). In addition, there were no significant correlations between lesion volume and any measure of recovery.

## Discussion

Overall, the findings from this study demonstrated that female monkeys evidenced less severe impairment of fine motor function of the hand and digits after a cortical injury in primary motor cortex than male monkeys even though the volume of the lesions did not significantly differ. Specifically, female monkeys showed less severe impairments early in the recovery period and a more complete recovery of grasp function than male monkeys. It is of interest to note that the female monkeys were significantly older than the male monkeys (mean age of females was 23.17 years and mean age of males was 16.81 years [F (1, 8) = 10.01, p = 0.0158] though they still showed a more complete recovery of function.

While a return to pre-injury levels of motor performance is rare in animal models and humans, it was of particular interest in the present study that four of the five female monkeys did return to a precise finger-thumb grasp rather than the compensatory grasp patterns observed in the male monkeys. While the development of a compensatory grasp pattern by the male monkeys did achieve the goal of retrieving the food reward on our motor task, this type of compensatory grasp, which is often observed in human stroke patients, is inefficient and does not translate into effective fine motor function of the digits that is required for successful completion of activities of daily living. Therefore, determining the underlying mechanisms of the less severe impairment and the greater degree of recovery of grasp pattern in the female monkeys is of critical importance.

### Sex differences in the pathogenesis of cortical injury

The pathogenesis of cortical injury is characterized by a cascade initiated by acute cellular damage, eventually leading to a sustained inflammatory response, chronic accumulation of oxidative stress, and secondary damage, which impairs cortical reorganization and recovery [68–71]. The brain inflammatory cells, microglia and astrocytes, release several cytotoxic agents including matrix metalloproteinases, nitric oxide, and reactive oxygen species (ROS) which lead to cell death [70, 72–74]. The immune response following cortical injury involves increases in reactive astrocytes and microglia that produce ROS and inflammatory cytokines and chemokines [69–71, 73, 75], which can also disrupt neuronal recovery and reorganization.

A large body of evidence from studies in rodents demonstrates sex differences in these immune responses to cortical injury [13, 22, 24, 39, 40, 76]. In rodents, a diminished pro-inflammatory response associated with a lower density of amoeboid (activated) microglia was found in females compared to males after recovery from ischemic injury [77–79]. Additionally, male neurons and astrocytes are more sensitive to ischemia and oxygen–glucose deprivation than female cells in vitro [76, 80–82]. In response to the inflammatory agent, lipopolysaccharide, male astrocytes show enhanced expression of pro-inflammatory cytokines, IL6, TNF-alpha and IL1B, while female astrocytes show enhanced expression of the anti-inflammatory cytokine, IL10 [83, 84]. Further, there is decreased ROS production in astrocytes in female brains compared to male brains [85–87]. In addition to these inflammatory responses, studies with cell cultures have demonstrated sex specific neuronal death mechanisms. For instance, cell death after ischemia in primary neuronal cultures derived from male brains are mediated by excessive ROS production and over-activation of poly(ADP)ribose polymerase (PARP) while death of cells from female brains involves programmed caspase-dependent apoptosis [43, 88–92].

One potential mechanism underlying age-dependent sex differences is the release of estradiol following brain injury. As a neuroprotective agent, estradiol acts on ER-α and ER-β receptors expressed by neurons, astrocytes, and microglia to modulate inflammation and promote plasticity [13, 15, 22, 24, 39, 93]. Acute 17ß-E2 treatment increases spine density and stabilization of newly formed spines through recruitment of synaptic proteins and receptors in cultured cortical neurons [19]. In rodent and monkey studies, estradiol treatment has shown to play an important role in rescuing age-related synaptic plasticity by modulating actin and synapse formation [94–96]. In addition, estradiol stimulates neuronal survival by altering the expression of the anti-apoptotic gene, *bcl-2* that inhibits free radical formation [24]. Further, estradiol can reduce inflammation through interactions with neurotrophic factors and by directly acting on the ER-α receptors on astrocytes and microglia [13, 39, 40, 85, 97–99]. Astrocytes express estrogen receptors and produce 17ß-E2 in both males and females [25, 99, 100]. Following cortical injury, astrocyte-derived 17ß-E2 mediates anti-inflammatory effects through the release of neurotrophic factors, BDNF, IGF-1, and GLT-1, as the astrocytes become reactive and increase expression of ER-α receptors and glial fibrillary acidic protein (GFAP) [9, 25, 93, 97, 101–105].

### Estradiol levels in aged rhesus monkeys

While there is substantial evidence that acute estradiol treatment in vitro and increased estradiol after injury in vivo in rodents leads to neuroprotection, how the cyclical changes in estradiol influence susceptibility to injury is not clear. There are varied definitions of menopause in humans, ranging from permanent cessation of menstruation to cessation of steroid hormone secretion [106, 107]. However, the most commonly considered criterion for menopause is the permanent, non-pathologic, age-associated cessation of ovulation as measured by increases in follicle stimulating hormone (FSH) coupled with decreases in anti-Mullerian hormone (AMH), inhibin B, and estradiol [108, 109]. Using this criterion, most studies place the mean age of menopause in humans at approximately 50 years of age. Studies of female rhesus monkeys have shown ovarian and hormonal changes that parallel humans which has led to age related characterization of both perimenopausal and menopausal stages leading to reproductive senescence [110, 111]. Current estimates for the age of onset of menopause in captive rhesus monkeys range from 22–27 years of age [110–113]. The monkeys in the current study ranged from 20–26 years of age and were therefore likely undergoing age-related reductions in FSH, AMH, inhibin B, and estradiol [106, 110, 114, 115]. While we did not monitor post-operative estradiol levels during the recovery period of the present study, it will be important for future investigations to assess whether increases in estradiol levels induced by injury may facilitate recovery.

### Sex differences in human stroke and recovery

While our model does not replicate stroke per se, it does model the injury and inflammatory cascade that occurs following stroke and therefore provides insight about recovery of function, plasticity, and repair in the brain and potential sex differences in these recovery processes. Additionally, stroke that occurs in humans is typically not isolated to the motor cortex, however, the focal lesion produced in our model provides an important means of quantifying and measuring recovery. Regarding sex differences in humans, there is a higher incidence of stroke in males until the age of 65 years. Afterwards, the prevalence and severity of stroke among females significantly increases [2, 4–6, 8, 116]. This shift during advanced age is attributed to decreases in estrogens and its corresponding neuroprotective effects, especially 17ß-E2 [9].

Clinical studies demonstrate considerable evidence that post-menopausal females experience greater stroke severity than their male counterparts, but differences in functional recovery remain unclear. For example, it has been shown that males experience a greater relative loss of muscle strength than females when post-stroke upper extremity muscle strength in the affected limb is reported as a percentage of strength of the unaffected side [117]. However, most studies demonstrate an overall lesser degree of physical recovery in females [2, 4, 116, 118–121]. The degree of recovery after stroke or other brain injuries is often assessed in large clinical trials by evaluating activities of daily living (ADL) using the Barthel Index (BI) or the modified Rankin scale (mRS) [122]. Both scales are widely used, but are frequently challenged by questions of subjectivity, reliability, and sensitivity [122, 123]. In addition, the scales do not assess the level of return to pre-injury motor function, but instead only measure independence level while performing daily tasks or routines. Since males typically experience stroke at a younger age than females, the difference in scores is often explained by males having more assistance with ADLs from a spouse, while females are more likely to be widowed at the time they experience a stroke [2, 120, 124]. Thus, the independence levels reported by women are often lower and associated with less assistance at home to perform their tasks [124]. Clinical studies are further challenged by other social and biological factors that include confounding factors such as mental health, lifestyle, comorbid risk factors (i.e., cardiovascular disease, diabetes mellitus, hypertension, etc.), household expectations, and family support [4, 6].

### Perspectives and significance

Currently, there is no consensus on outcome measures that can provide a more complete and quantitative assessment of post-stroke recovery in human females compared to males [125]. Despite the clinical and epidemiological evidence of sex differences, the variability and confounding factors inherent to clinical studies affirms the importance of translatable models that can provide quantitative analyses of functional impairment and recovery after injury.

## Conclusions

Overall, female monkeys in this study showed less severe impairment and more complete recovery than male monkeys even though there were no significant differences in the lesion volume. Due to the age of the female monkeys and observed individual differences in measures of recovery, it is possible that differences in recovery were due to hormonal factors such as estradiol and progesterone which are involved in providing neuroprotection in the female brain [32, 33, 126, 127]. A complete explanation of the present results of reduced severity of impairment and enhanced recovery of function in females will require more mechanistic studies with the use of estradiol supplementation constituting one important avenue for future investigation.

## Data Availability

All data generated and/or analyzed during this study are included in this published article.

## References

[1] Seshadri S, Beiser A, Kelly-Hayes M, Kase CS, Au R, Kannel WB (2006). The lifetime risk of stroke: estimates from the Framingham Study. Stroke.

[2] Petrea RE, Beiser AS, Seshadri S, Kelly-Hayes M, Kase CS, Wolf PA (2009). Gender differences in stroke incidence and poststroke disability in the Framingham Heart Study. Stroke.

[3] Andersen MN, Andersen KK, Kammersgaard LP, Olsen TS (2005). Sex differences in stroke survival: 10-year follow-up of the Copenhagen Stroke Study Cohort. J Stroke Cerebrovasc Dis.

[4] Lai S-M, Duncan PW, Dew P, Keighley J (2005). Sex differences in stroke recovery. Prev Chronic Dis.

[5] Appelros P, Stegmayr B, Terént A (2009). A review on sex differences in stroke treatment and outcome: sex differences in stroke treatment and outcome. Acta Neurol Scand.

[6] Fukuda M, Kanda T, Kamide N, Akutsu T, Sakai F (2009). Gender differences in long-term functional outcome after first-ever ischemic stroke. Intern Med.

[7] Manwani B, McCullough LD (2011). Sexual dimorphism in ischemic stroke: lessons from the laboratory. Womens Health (Lond Engl).

[8] Roger VL, Go AS, Lloyd-Jones DM, Adams RJ, Berry JD, Brown TM (2011). Heart disease and stroke statistics—2011 update: a report from the American Heart Association. Circulation.

[9] Schreihofer DA, Ma Y (2013). Estrogen receptors and ischemic neuroprotection: Who, what, where, and when?. Brain Res.

[10] Alkayed NJ, Harukuni I, Kimes AS, London ED, Traystman RJ, Hurn PD (1998). Gender-linked brain injury in experimental stroke. Stroke.

[11] Carswell HVO, Dominiczak AF, Macrae IM (2000). Estrogen status affects sensitivity to focal cerebral ischemia in stroke-prone spontaneously hypertensive rats. Am J Physiol Heart Circ Physiol.

[12] Simpkins JW, Green PS, Gridley KE, Singh M, de Fiebre NC, Rajakumar G (1997). Role of estrogen replacement therapy in memory enhancement and the prevention of neuronal loss associated with alzheimer’s disease. Am J Med.

[13] Dubal DB, Kashon ML, Pettigrew LC, Ren JM, Finklestein SP, Rau SW (1998). Estradiol Protects against Ischemic Injury. J Cereb Blood Flow Metab.

[14] Dubal DB, Wise PM (2001). Neuroprotective effects of estradiol in middle-aged female rats. Endocrinology.

[15] Brann DW, Dhandapani K, Wakade C, Mahesh VB, Khan MM (2007). Neurotrophic and neuroprotective actions of estrogen: basic mechanisms and clinical implications. Steroids.

[16] Guo J, Duckles SP, Weiss JH, Li X, Krause DN (2012). 17β-Estradiol prevents cell death and mitochondrial dysfunction by an estrogen receptor-dependent mechanism in astrocytes after oxygen-glucose deprivation/reperfusion. Free Radic Biol Med.

[17] Arevalo MA, Santos-Galindo M, Acaz-Fonseca E, Azcoitia I, Garcia-Segura LM (2013). Gonadal hormones and the control of reactive gliosis. Horm Behav.

[18] Sohrabji F, Bake S, Lewis DK (2013). Age-related changes in brain support cells: Implications for stroke severity. Neurochem Int.

[19] Sellers KJ, Erli F, Raval P, Watson IA, Chen D, Srivastava DP (2015). Rapid modulation of synaptogenesis and spinogenesis by 17β-estradiol in primary cortical neurons. Front Cell Neurosci.

[20] Wen Y, Yang S, Liu R, Perez E, Yi KD, Koulen P (2004). Estrogen attenuates nuclear factor-kappa B activation induced by transient cerebral ischemia. Brain Res.

[21] Bruce-Keller AJ, Keeling JL, Keller JN, Huang FF, Camondola S, Mattson MP (2000). Antiinflammatory effects of estrogen on microglial activation*. Endocrinology.

[22] Vegeto E, Belcredito S, Etteri S, Ghisletti S, Brusadelli A, Meda C (2003). Estrogen receptor-alpha mediates the brain antiinflammatory activity of estradiol. Proc Natl Acad Sci U S A.

[23] Santos RS, de Fatima LA, Frank AP, Carneiro EM, Clegg DJ (2017). The effects of 17 alpha-estradiol to inhibit inflammation in vitro. Biol Sex Differ.

[24] Dubal DB, Wilson ME, Wise PM (1999). Estradiol: a protective and trophic factor in the brain. J Alzheimers Dis.

[25] Lu Y, Sareddy GR, Wang J, Zhang Q, Tang F-L, Pratap UP (2020). Neuron-derived estrogen is critical for astrocyte activation and neuroprotection of the ischemic brain. J Neurosci.

[26] Guennoun R (2020). Progesterone in the brain: hormone, neurosteroid and neuroprotectant. Int J Mol Sci.

[27] Gibson CL, Gray LJ, Bath PMW, Murphy SP (2008). Progesterone for the treatment of experimental brain injury; a systematic review. Brain.

[28] Zárate S, Stevnsner T, Gredilla R (2017). Role of estrogen and other sex hormones in brain aging. Neuroprotection and DNA repair. Front Aging Neurosci..

[29] Yousuf S, Atif F, Sayeed I, Tang H, Stein DG (2014). Progesterone in transient ischemic stroke: a dose–response study. Psychopharmacology.

[30] Murphy SJ, Littleton-Kearney MT, Hurn PD (2002). Progesterone administration during reperfusion, but not preischemia alone, reduces injury in ovariectomized rats. J Cereb Blood Flow Metab.

[31] Gibson CL, Murphy SP (2004). Progesterone enhances functional recovery after middle cerebral artery occlusion in male mice. J Cereb Blood Flow Metab.

[32] Chen J, Chopp M, Li Y (1999). Neuroprotective effects of progesterone after transient middle cerebral artery occlusion in rat. J Neurol Sci.

[33] Jiang N, Chopp M, Stein D, Feit H (1996). Progesterone is neuroprotective after transient middle cerebral artery occlusion in male rats. Brain Res.

[34] Ishrat T, Sayeed I, Atif F, Stein DG (2009). Effects of progesterone administration on infarct volume and functional deficits following permanent focal cerebral ischemia in rats. Brain Res.

[35] Gaignard P, Fréchou M, Schumacher M, Thérond P, Mattern C, Slama A (2016). Progesterone reduces brain mitochondrial dysfunction after transient focal ischemia in male and female mice. J Cereb Blood Flow Metab.

[36] Guennoun R, Zhu X, Fréchou M, Gaignard P, Slama A, Liere P (2019). Steroids in stroke with special reference to progesterone. Cell Mol Neurobiol.

[37] Espinosa-Garcia C, Sayeed I, Yousuf S, Atif F, Sergeeva EG, Neigh GN (2017). Stress primes microglial polarization after global ischemia: therapeutic potential of progesterone. Brain Behav Immun.

[38] Gibson CL, Constantin D, Prior MJW, Bath PMW, Murphy SP (2005). Progesterone suppresses the inflammatory response and nitric oxide synthase-2 expression following cerebral ischemia. Exp Neurol.

[39] Wise PM, Dubal DB, Wilson ME, Rau SW, Liu Y (2001). Estrogens: trophic and protective factors in the adult brain. Front Neuroendocrinol.

[40] Vegeto E, Belcredito S, Ghisletti S, Meda C, Etteri S, Maggi A (2006). The endogenous estrogen status regulates microglia reactivity in animal models of neuroinflammation. Endocrinology.

[41] Kreutzberg GW (1996). Microglia: a sensor for pathological events in the CNS. Trends Neurosci.

[42] Haley PJ (2003). Species differences in the structure and function of the immune system. Toxicology.

[43] Du L, Bayir H, Lai Y, Zhang X, Kochanek PM, Watkins SC (2004). Innate gender-based proclivity in response to cytotoxicity and programmed cell death pathway. J Biol Chem.

[44] Brotfain E, Gruenbaum SE, Boyko M, Kutz R, Zlotnik A, Klein M (2016). Neuroprotection by estrogen and progesterone in traumatic brain injury and spinal cord injury. Curr Neuropharmacol.

[45] Siddiqui AN, Siddiqui N, Khan RA, Kalam A, Jabir NR, Kamal MA (2016). Neuroprotective role of steroidal sex hormones: an overview. CNS Neurosci Ther.

[46] Céspedes Rubio ÁE, Pérez-Alvarez MJ, Lapuente Chala C, Wandosell F (2018). Sex steroid hormones as neuroprotective elements in ischemia models. J Endocrinol.

[47] Moore TL, Killiany RJ, Pessina MA, Moss MB, Finklestein SP, Rosene DL (2012). Recovery from ischemia in the middle-aged brain: a nonhuman primate model. Neurobiol Aging.

[48] Moore TL, Pessina MA, Finklestein SP, Kramer BC, Killiany RJ, Rosene DL (2013). Recovery of fine motor performance after ischemic damage to motor cortex is facilitated by cell therapy in the rhesus monkey. Somatosens Mot Res.

[49] Moore TL, Pessina MA, Finklestein SP, Killiany RJ, Bowley B, Benowitz L (2016). Inosine enhances recovery of grasp following cortical injury to the primary motor cortex of the rhesus monkey. Restor Neurol Neurosci.

[50] Moore TL, Bowley BGE, Shultz PL, Calderazzo SM, Shobin EJ, Uprety AR (2018). Oral curcumin supplementation improves fine motor function in the middle-aged rhesus monkey. Somatosens Mot Resh..

[51] Moore TL, Bowley BGE, Pessina MA, Calderazzo SM, Medalla M, Go V (2019). Mesenchymal derived exosomes enhance recovery of motor function in a monkey model of cortical injury. Restor Neurol Neurosci.

[52] Tigges J, Gordon TP, McClure HM, Hall EC, Peters A (1988). Survival rate and life span of rhesus monkeys at the Yerkes regional primate research center. Am J Primatol.

[53] Moore TL, Killiany RJ, Pessina MA, Moss MB, Rosene DL (2010). Assessment of motor function of the hand in aged rhesus monkeys. Somatosens Mot Res.

[54] Kluver H (1935). An auto-multi-stimulation reaction board for use with sub-human primates. J Psychol.

[55] Corbetta M, Ramsey L, Callejas A, Baldassarre A, Hacker CD, Siegel JS (2015). Common behavioral clusters and subcortical anatomy in stroke. Neuron.

[56] Souza WC, Conforto AB, Orsini M, Stern A, André C (2015). Similar effects of two modified constraint-induced therapy protocols on motor impairment, motor function and quality of life in patients with chronic stroke. Neurol Int.

[57] Kwakkel G, Winters C, van Wegen EEH, Nijland RHM, van Kuijk AAA, Visser-Meily A (2016). Effects of unilateral upper limb training in two distinct prognostic groups early after stroke: the EXPLICIT-stroke randomized clinical trial. Neurorehabil Neural Repair.

[58] Hylin MJ, Kerr AL, Holden R (2017). Understanding the mechanisms of recovery and/or compensation following injury. Neural Plast.

[59] Levin MF, Kleim JA, Wolf SL (2009). What do motor “recovery” and “compensation” mean in patients following stroke?. Neurorehabil Neural Repair.

[60] Lum PS, Mulroy S, Amdur RL, Requejo P, Prilutsky BI, Dromerick AW (2009). Gains in upper extremity function after stroke via recovery or compensation: potential differential effects on amount of real-world limb use. Top Stroke Rehabil.

[61] Pessina MA, Bowley BGE, Rosene DL, Moore TL (2019). A method for assessing recovery of fine motor function of the hand in a rhesus monkey model of cortical injury: an adaptation of the Fugl-Meyer Scale and Eshkol-Wachman Movement Notation. Somatosens Mot Res.

[62] Carr JH, Shepherd RB, Nordholm L, Lynne D (1985). Investigation of a new motor assessment scale for stroke patients. Phys Ther.

[63] Whishaw IQ, Suchowersky O, Davis L, Sarna J, Metz GA, Pellis SM (2002). Impairment of pronation, supination, and body co-ordination in reach-to-grasp tasks in human Parkinson’s disease (PD) reveals homology to deficits in animal models. Behav Brain Res.

[64] Fugl-Meyer AR, Jääskö L, Leyman I, Olsson S, Steglind S (1975). The post-stroke hemiplegic patient. 1. a method for evaluation of physical performance. Scand J Rehabil Med.

[65] Rosene DL, Roy NJ, Davis BJ (1986). A cryoprotection method that facilitates cutting frozen sections of whole monkey brains for histological and histochemical processing without freezing artifact. J Histochem Cytochem.

[66] Estrada LI, Robinson AA, Amaral AC, Giannaris EL, Heyworth NC, Mortazavi F (2017). Evaluation of long-term cryostorage of brain tissue sections for quantitative histochemistry. J Histochem Cytochem.

[67] Go V, Bowley BGE, Pessina MA, Zhang ZG, Chopp M, Finklestein SP (2019). Extracellular vesicles from mesenchymal stem cells reduce microglial-mediated neuroinflammation after cortical injury in aged Rhesus monkeys. Geroscience.

[68] Flamm ES, Demopoulos HB, Seligman ML, Poser RG, Ransohoff J (1978). Free radicals in cerebral ischemia. Stroke.

[69] Taylor JM, Crack PJ (2004). Impact of oxidative stress on neuronal survival. Clin Exp Pharmacol Physiol.

[70] Wang Q, Tang X, Yenari M (2007). The inflammatory response in stroke. J Neuroimmunol.

[71] Lakhan SE, Kirchgessner A, Hofer M (2009). Inflammatory mechanisms in ischemic stroke: therapeutic approaches. J Transl Med.

[72] Sugawara T, Chan PH (2003). Reactive oxygen radicals and pathogenesis of neuronal death after cerebral ischemia. Antioxid Redox Signal.

[73] Margaill I, Plotkine M, Lerouet D (2005). Antioxidant strategies in the treatment of stroke. Free Radical Biol Med.

[74] Patel AR, Ritzel R, McCullough LD, Liu F (2013). Microglia and ischemic stroke: a double-edged sword. Int J Physiol Pathophysiol Pharmacol.

[75] Warner DS (2004). Oxidants, antioxidants and the ischemic brain. J Exp Biol.

[76] Liu M, Hurn PD, Roselli CE, Alkayed NJ (2007). Role of P450 aromatase in sex-specific astrocytic cell death. J Cereb Blood Flow Metab.

[77] Xiong X, Xu L, Wei L, White RE, Ouyang Y-B, Giffard RG (2015). IL-4 is required for sex differences in vulnerability to focal ischemia in mice. Stroke.

[78] Hanamsagar R, Bilbo SD (2016). Sex differences in neurodevelopmental and neurodegenerative disorders: Focus on microglial function and neuroinflammation during development. J Steroid Biochem Mol Biol.

[79] McCarthy MM, Nugent BM, Lenz KM (2017). Neuroimmunology and neuroepigenetics in the establishment of sex differences in the brain. Nat Rev Neurosci.

[80] Heyer A, Hasselblatt M, von Ahsen N, Häfner H, Sirén A-L, Ehrenreich H (2005). In vitro gender differences in neuronal survival on hypoxia and in 17beta-estradiol-mediated neuroprotection. J Cereb Blood Flow Metab.

[81] Liu M, Oyarzabal EA, Yang R, Murphy SJ, Hurn PD (2008). A novel method for assessing sex-specific and genotype-specific response to injury in astrocyte culture. J Neurosci Methods.

[82] Johnsen D, Murphy SJ (2011). Isoflurane preconditioning protects astrocytes from oxygen and glucose deprivation independent of innate cell sex. J Neurosurg Anesthesiol.

[83] Santos-Galindo M, Acaz-Fonseca E, Bellini MJ, Garcia-Segura LM (2011). Sex differences in the inflammatory response of primary astrocytes to lipopolysaccharide. Biol Sex Differ.

[84] Loram LC, Sholar PW, Taylor FR, Wiesler JL, Babb JA, Strand KA (2012). Sex and estradiol influence glial pro-inflammatory responses to lipopolysaccharide in rats. Psychoneuroendocrinology.

[85] Cordeau P, Lalancette-Hébert M, Weng YC, Kriz J (2008). Live imaging of neuroinflammation reveals sex and estrogen effects on astrocyte response to ischemic injury. Stroke.

[86] Sohrabji F, Williams M (2013). Stroke neuroprotection: oestrogen and insulin-like growth factor-1 interactions and the role of microglia. J Neuroendocrinol.

[87] Morrison HW, Filosa JA (2016). Sex differences in astrocyte and microglia responses immediately following middle cerebral artery occlusion in adult mice. Neuroscience.

[88] Lang JT, McCullough LD (2008). Pathways to ischemic neuronal cell death: are sex differences relevant?. J Transl Med.

[89] Yuan M, Siegel C, Zeng Z, Li J, Liu F, McCullough LD (2009). Sex differences in the response to activation of the poly (ADP-ribose) polymerase pathway after experimental stroke. Exp Neurol.

[90] Vagnerova K, Liu K, Ardeshiri A, Cheng J, Murphy SJ, Hurn PD (2010). PARP-1 initiated neuronal cell death pathway–do androgens matter?. Neuroscience.

[91] Jia J, Verma S, Nakayama S, Quillinan N, Grafe MR, Hurn PD (2011). Sex differences in neuroprotection provided by inhibition of TRPM2 channels following experimental stroke. J Cereb Blood Flow Metab.

[92] Herson PS, Palmateer J, Hurn PD (2013). Biological sex and mechanisms of ischemic brain injury. Transl Stroke Res.

[93] Zhang Q-G, Wang R, Tang H, Dong Y, Chan A, Sareddy GR (2014). Brain-derived estrogen exerts anti-inflammatory and neuroprotective actions in the rat hippocampus. Mol Cell Endocrinol.

[94] Wang ACJ, Hara Y, Janssen WGM, Rapp PR, Morrison JH (2010). Synaptic estrogen receptor-α levels in prefrontal cortex in female rhesus monkeys and their correlation with cognitive performance. J Neurosci.

[95] Hara Y, Waters EM, McEwen BS, Morrison JH (2015). Estrogen effects on cognitive and synaptic health over the lifecourse. Physiol Rev.

[96] Beckman D, Ott S, Donis-Cox K, Janssen WG, Bliss-Moreau E, Rudebeck PH (2019). Oligomeric Aβ in the monkey brain impacts synaptic integrity and induces accelerated cortical aging. Proc Natl Acad Sci USA.

[97] Arevalo MA, Santos-Galindo M, Lagunas N, Azcoitia I, Garcia-Segura LM (2011). Selective estrogen receptor modulators as brain therapeutic agents. J Mol Endocrinol.

[98] Zuo W, Zhang W, Chen N-H (2013). Sexual dimorphism in cerebral ischemia injury. Eur J Pharmacol.

[99] Chisholm NC, Sohrabji F (2016). Astrocytic response to cerebral ischemia is influenced by sex differences and impaired by aging. Neurobiol Dis.

[100] Zwain IH, Yen SSC (1999). Neurosteroidogenesis in astrocytes, oligodendrocytes, and neurons of cerebral cortex of rat brain. Endocrinology.

[101] Blurton-Jones M, Tuszynski MH (2001). Reactive astrocytes express estrogen receptors in the injured primate brain. J Comp Neurol.

[102] Saldanha CJ, Duncan KA, Walters BJ (2009). Neuroprotective actions of brain aromatase. Front Neuroendocrinol.

[103] Azcoitia I, Santos-Galindo M, Arevalo MA, Garcia-Segura LM (2010). Role of astroglia in the neuroplastic and neuroprotective actions of estradiol: estradiol and astroglia. Eur J Neurosci.

[104] Barreto GE, Santos-Galindo M, Garcia-Segura LM (2014). Selective estrogen receptor modulators regulate reactive microglia after penetrating brain injury. Front Aging Neurosci.

[105] Ma Y, Guo H, Zhang L, Tao L, Yin A, Liu Z (2016). Estrogen replacement therapy-induced neuroprotection against brain ischemia-reperfusion injury involves the activation of astrocytes via estrogen receptor β. Sci Rep.

[106] Walker ML, Herndon JG (2008). Menopause in nonhuman primates?. Biol Reprod.

[107] Takahashi TA, Johnson KM (2015). Menopause. Med Clin North Am.

[108] Su HI, Freeman EW (2009). Hormone changes associated with the menopausal transition. Minerva Ginecol.

[109] Santoro N, Randolph JF (2011). Reproductive hormones and the menopause transition. Obstet Gynecol Clin North Am.

[110] Walker ML (1995). Menopause in female rhesus monkeys. Am J Primatol.

[111] Gilardi KVK, Shideler SE, Valverde CR, Roberts JA, Lasley BL (1997). Characterization of the onset of menopause in the rhesus macaque1. Biol Reprod.

[112] Keverne EB, Michael RP (1970). Annual changes in the menstruation of rhesus monkeys. J Endocrinol.

[113] Van Wagenen G (1972). Vital statistics from a breeding colony. Reproduction and pregnancy outcome in Macaca mulatta. J Med Primatol.

[114] Hotchkiss J, Atkinson LE, Knobile E (1971). Time course of serum estrogen and luteinizing hormone (LH) concentrations during the menstrual cycle of the rhesus monkey. Endocrinology.

[115] Bellino FL, Wise PM (2003). Nonhuman primate models of menopause workshop1. Biol Reprod.

[116] Gargano JW, Reeves MJ, Paul Coverdell National Acute Stroke Registry Michigan Prototype Investigators (2007). Sex differences in stroke recovery and stroke-specific quality of life: results from a statewide stroke registry. Stroke.

[117] Starosta M, Miller E, Redlicka J, Kostka J (2017). Analysis of upper limb muscle strength in the early phase of brain stroke. Acta Bioeng Biomech.

[118] Di Carlo A, Lamassa M, Baldereschi M, Pracucci G, Basile AM, Wolfe CDA (2003). Sex differences in the clinical presentation, resource use, and 3-month outcome of acute stroke in Europe: data from a multicenter multinational hospital-based registry. Stroke.

[119] Kapral MK, Fang J, Hill MD, Silver F, Richards J, Jaigobin C (2005). Sex differences in stroke care and outcomes: results from the Registry of the Canadian Stroke Network. Stroke.

[120] Reeves MJ, Bushnell CD, Howard G, Gargano JW, Duncan PW, Lynch G (2008). Sex differences in stroke: epidemiology, clinical presentation, medical care, and outcomes. Lancet Neurol.

[121] Kim J-S, Lee K-B, Roh H, Ahn M-Y, Hwang H-W (2010). Gender differences in the functional recovery after acute stroke. J Clin Neurol.

[122] Quinn TJ, Langhorne P, Stott DJ (2011). Barthel index for stroke trials: development, properties, and application. Stroke.

[123] Sreekrishnan A, Leasure AC, Shi F-D, Hwang DY, Schindler JL, Petersen NH (2017). Functional improvement among intracerebral hemorrhage (ICH) survivors up to 12 months post-injury. Neurocrit Care.

[124] Sue-Min L, Duncan PW, Dew P, Keighley J (2005). Sex differences in stroke recovery. Prev Chronic Dis.

[125] Baker K, Cano SJ, Playford ED (2011). Outcome measurement in stroke: a scale selection strategy. Stroke.

[126] Cervantes M, González-Vidal MD, Ruelas R, Escobar A, Moralí G. Neuroprotective effects of progesterone on damage elicited by acute global cerebral ischemia in neurons of the caudate nucleus. Arch Med Res 2002;33(1):6-14. 10.1016/s0188-4409(01)00347-2 11825624

[127] Xu F-F, Sun S, Ho ASW, Lee D, Kiang KMY, Zhang X-Q (2014). Effects of progesterone vs. dexamethasone on brain oedema and inflammatory responses following experimental brain resection. Brain Inj.

